# Manual Material Handling Training: The Effect of Self-Observation, Hetero-Observational and Intrinsic Feedback on Workers’ Knowledge and Behaviour

**DOI:** 10.3390/ijerph17218095

**Published:** 2020-11-03

**Authors:** Anna M. Sene-Mir, Mariona Portell, M. Teresa Anguera, Salvador Chacón-Moscoso

**Affiliations:** 1 Physical Activity and Sports Studies Centre, University of Vic-Central University of Catalonia, 08500 Vic, Barcelona, Spain; annam.sene@uvic.cat; 2 Department of Psychobiology and Methodology in Health Sciences, Universitat Autònoma de Barcelona, Cerdanyola del Vallès, 08193 Barcelona, Spain; 3 Faculty of Psychology, Institute of Neurosciences, University of Barcelona, 08035 Barcelona, Spain; mtanguera@gmail.com; 4 Departamento de Psicología Experimental, Universidad de Sevilla, 41018 Seville, Spain; schacon@us.es; 5 Departamento de Psicología, Universidad Autónoma de Chile, Santiago 7500138, Chile

**Keywords:** observation, self-observation, hetero-observation, feedback, feedforward, manual material handling, knowledge, behaviour, workplace intervention, SsObserWork

## Abstract

This study aimed to assess the effect of systematic self-observation, hetero-observational feedback, and feedforward and intrinsic feedback (SsObserWork components) on workers’ knowledge and behaviour of a manual material handling (MMH) technique in the industrial sector. Blue-collar workers recruited from a food processing company in Catalonia (Spain) were randomized into SsObserWork (*N* = 31) and control (*N* = 30) groups. SsObserWork group members participated individually in two sessions and a three-week follow-up between sessions where they received the SsObserWork components. The control group participated individually in two sessions where they received a standard MMH training. An ad hoc instrumentcalled the MMH-SsObserWork instrument was used to assess the MMH behaviour, and an adaption of the instrument was done to assess the workers’ knowledge. Significant differences were found between groups for the identification of recommended back positions in the first session and also on comparing both sessions. However, no differences were found for the rest of the criteria. There also were significant differences between groups in the score changes of the back, knee joints, elbow joints, and interaction criterion, indicating that the SsObserWork group improved the MMH performance in these criteria (behaviour). SsObserWork intervention showed a positive effect on improving the knowledge and behaviour of the MMH technique, specifically on back posture.

## 1. Introduction

Manual handling has been associated with an increased risk of back disorders [1], mainly lower back pain [2]. In the industrial sector, most tasks have been automated, although manual handling is still being carried out when it cannot be avoided. Furthermore, people are not only exposed to manual handling at work, but there also are many situations in daily life where it is required and can increase the risk of back disorders. Hence, European Union directives, such as Council Directive 90/269/EEC and national legislations of member states have been outlining appropriate MMH training [3]. Considering the requirement for MMH training in workplaces, numerous studies have been focused on developing, implementing, and evaluating the efficacy of manual handling training on reduction of lower back pain or back injury prevention [4]. It has been observed that fewer studies have been conducted on the effectiveness of manual handling training in industries outside of the healthcare sector [5].

Different systematic reviews have concluded that the majority of manual handling training procedures are not effective to reduce lower back pain or back injury [5]. This lack of effectiveness is associated with one-dimensional interventions, lack of transferability of training, and a lack of training based on the theory of changing health behaviour. Moreover, studies tend to evaluate the effectiveness of training on long-term results, such as reduction in musculoskeletal disorders [5,6], and have omitted intermediate variables. Hogan et al. [7] pointed out that there is the need to evaluate the effect of manual handling training on intermediate variables, such as knowledge, behaviour change, and training transferability. To date, few studies have evaluated the effect of MMH training on knowledge and behaviour change [5,6].

In the last decades, studies focused on MMH training have evaluated different training methods. They tended to combine lifting training with back school, lumbar support, verbal feedback, practice, biofeedback, warm-up exercise, and ergonomic redesign of equipment. In addition, physical exercise has been highlighted as a component of MMH training [5] because it helps to improve leg strength and consequently avoid back loading [8].

However, one of the current challenges is how to actively involve employees in the training, taking into account that the higher the involvement in the training, the more effective it will be [9]. In this sense, a training method that could emphasize the active role of the employee in the training is that of self-observation. Self-observation is a relevant method to promote behaviour change, because it influences self-confidence, self-awareness, and self-efficacy, and has an emotional impact [10,11]. Self-observation has been evaluated in interventions focused on behaviour change, such as in the treatment of attention deficit hyperactivity disorder, autism, emotional disturbances, speaking ability, and so on [12], and for skill acquisition in sport [13,14]. Moreover, self-observation has been applied in different training programs to improve the interaction skills of professionals [15], and to increase one’s own teaching [16]. Nonetheless, there is little evidence of self-observation as a formative method in training for risk prevention and health promotion at work, and specifically in MMH training. As far as we know, self-observation (specifically self-modelling) has been applied as a training method for reducing musculoskeletal risk among office workers using computers, showing positive effects [17]. It has also been implemented to improve movement awareness among nursing students, who highlighted its usefulness and its cognitive impact with regard to changing body postures during patient transfer [18,19]. In all these cases, self-observation was done through video.

In the learning process of motor activities (e.g., MMH technique), video self-observation can provide two types of external feedback: knowledge of results and knowledge of performance. Usually, video self-observation tends to be used to give knowledge of performance [20]. However, video self-observation can provide a lot of information that could have a negative effect on employee attention. This is why video self-observation should be complemented with external feedback from the technician, who should give transitional information that reports what is well done [21], what should be improved, and how it should be done—also called feedforward [10]. We call this type of external feedback hetero-observational feedback and feedforward. Even so, it is important to consider that the employee will not always receive these external feedbacks because the training takes a particular period of time. Therefore, the training should also promote the use of intrinsic feedback (information provided from intrinsic sources, such as vision and proprioception) with the proposal of making the employees aware of their movements and positions adopted. Schmidt and Lee [20] suggested that it is this intrinsic feedback that one must learn to interpret, because it will always be available to the learner. Thus, by promoting intrinsic feedback, employees will be capable of regulating themselves once the training has finished.

Additionally, another way to conduct self-observation in a natural environment is through ambulatory assessment (e.g., using a self-report questionnaire) [22,23]. Studies have shown that ambulatory assessment as an intervention component promotes self-awareness and behavioural change [19,24]. However, it is important to take into account that these behaviour changes can also be caused by reactivity [22,25].

The positive effects of self-observation, hetero-observational and intrinsic feedbacks have been widely studied in sport skill acquisition [20,25,26], even though there is a lack of studies on risk prevention and health promotion.

Therefore, the aim of this study was to assess the effect of the previous components on worker’s knowledge and behaviour of the MMH technique by employing a randomised controlled trial. We use the name SsObserWork to identify the intervention. Specifically, we tested and evaluated the effect of the main components of SsObserWork, which are systematic self-observation (SSO), hetero-observational feedback and feedforward (HFF), and intrinsic feedback. It was hypothesized that these three main components improved employee knowledge and behaviour of the MMH technique.

## 2. Materials and Methods

### 2.1. Study Design

A parallel randomised trial of two groups was conducted among blue-collar workers recruited from a food processing company in Catalonia, Spain. Participants were randomly allocated to the SsObserWork or control groups. To evaluate the intervention effect, we adopted a methodological complementarity perspective [27], combining experimental design with observational methodology. Observational methodology is essential in this study in order to evaluate behaviour, to implement the self-observation component, and to plan the data collection [28,29,30,31]. In this sense, according to observational methodology, the study adopted a follow-up, nomothetic, and multidimensional design [28].

The Ethics Committee of the Autonomous University of Barcelona approved the study protocol (PRO1742). Prior to commencement of the intervention, the participants were made aware of their right to withdraw from the study at any time and steps to safeguard information. In accordance with the principles of the Declaration of Helsinki, the participants were informed that they were being recorded. They were shown the location of the video cameras, which were positioned directly to minimize reactivity bias. Informed consent was also obtained.

### 2.2. Participants and Procedure

**Figure 1:**
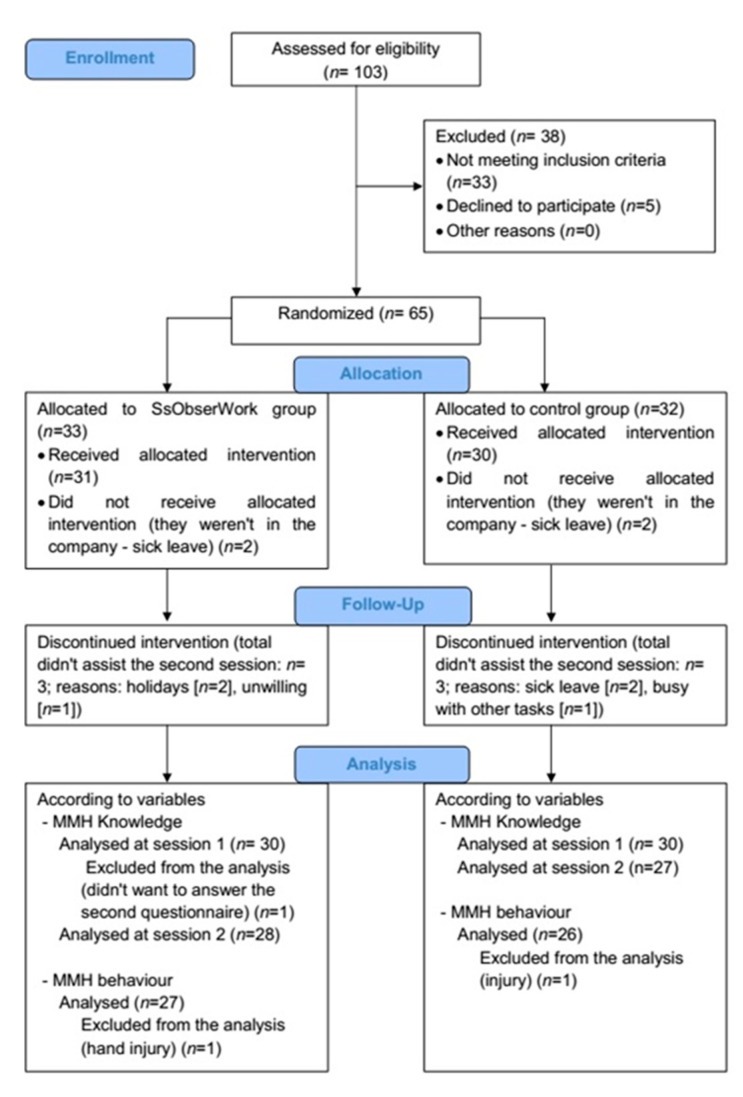
Consort flow diagram.

We used a nonprobability sampling. A convenience sample was initially composed of 103 blue-collar workers at a leading company in the Spanish meat sector, certified by the food safety regulations of the International Food Standards (IFS) and the British Retail Consortium (BRC). Completing the questionnaire and participating in the study was voluntary. The questionnaires were collected during September and October 2015. From the first sample, purposive sampling was applied, using the information provided in the questionnaire. The inclusion criteria were (i) to be older than 18 years old, (ii) to be employed as a blue-collar worker, (iii) not suffer any chronic bone, muscle, or joint disease in the trunk, and/or chronic or acute pain diagnosed by a specialist, and (iv) not suffer any chronic or acute knee joint disease diagnosed by a specialist. Of the 103 employees who completed the questionnaire, 65 were eligible and willing to participate. The 65 participants were randomly assigned to the SsObserWork or control group (Figure 1).

### 2.3. Intervention

The SsObserWork intervention was designed taking into consideration the Health Belief Model [32] and trans-theoretical model of behaviour change [33]. The aim of the intervention was to raise awareness and promote the adoption of proper postural back habits during MMH tasks, considered as complex tasks and a risk factor to develop lower back pain [2]. We considered that by adopting a proper bask posture during MMH, it could be transferred to other working and daily life tasks.

**Figure 2:**
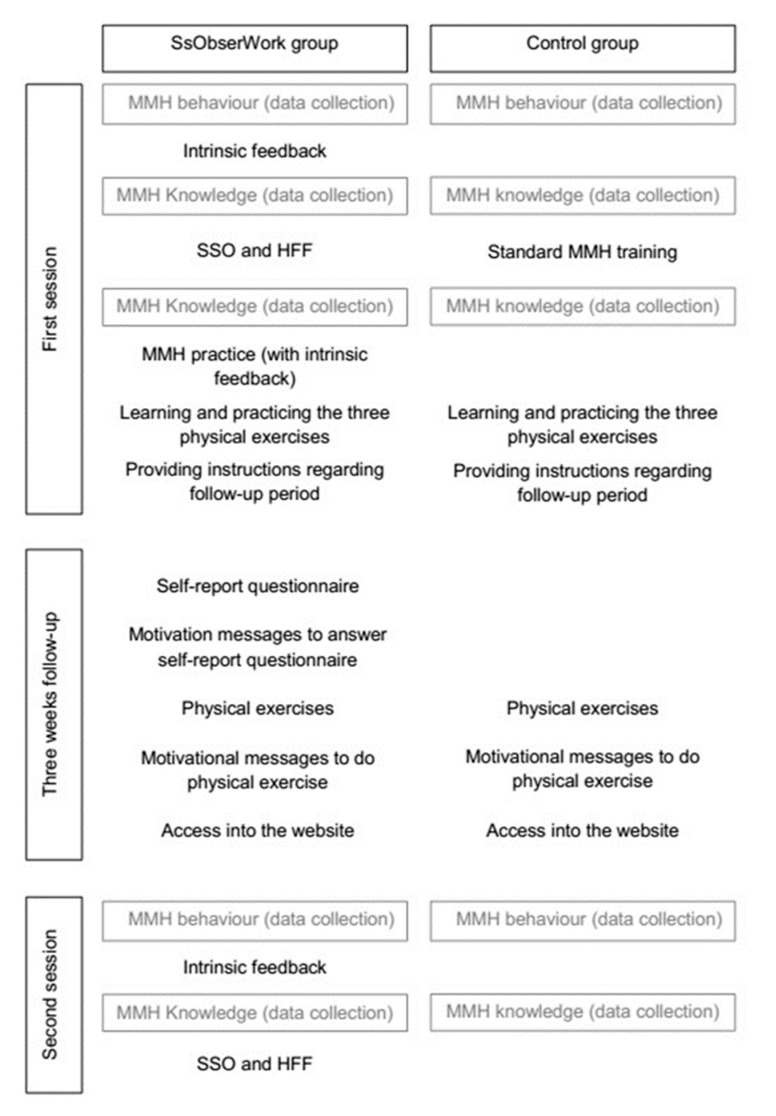
Intervention structure with data collection according to SsObserWork and control groups.

The SsObserWork intervention was made up of components, formative activities, and didactic materials, which are described in Appendix A. The intervention consisted of two sessions and a three-week follow-up between sessions. The intervention was implemented by a trained physical activity specialist. Figure 2 shows the intervention structure and how the components, formative activities, didactic materials, and data collection were distributed orderly. As shown, SSO (including self-report questionnaire), HFF, and intrinsic feedback are the components that evaluated their effect. Derivatively, MMH practice (which promotes intrinsic feedback) and messages to remind workers to answer the self-report questionnaire were only implemented in the SsObserWork group. Instead of the SsObserWork components, the control group received a standard MMH training based on theoretical information. The theoretical information consisted of explaining how to perform a MMH task and without any type of practice. Each SsObserWork and control session was carried out individually with each participant during November and December 2015.

### 2.4. Measures

Baseline characteristics of participants. An ad hoc questionnaire was made to select the sample. It consisted of demographic, social, and health questions, and it included the Standardised Nordic Questionnaire [34], SF-12v2 Health Survey [35], and the Utrecht Work Engagement Scale [36] and stages-of-change Items [37].

MMH behaviour: MMH behaviour was measured with an ad hoc instrument called the MMH-SsObserWork instrument. This observational instrument allows to identify and describe the body positions adopted continually during MMH tasks by using a set of criteria and categories. The criteria are: feet, knee joint, back, elbow joint, load position, and interaction between back tilt and move around. At the beginning of each session, workers had to lift, carry, and lower five boxes (8 kg each) while they were recorded from the sagittal plane. The video camera was positioned at the height of the worker’s hip. The MMH-SsObserWork instrument had to be used using a software application for the record and computation of observational data (e.g., Lince) in which the video recording can be displayed. The MMH-SsObserWork instrument has a very good inter-observer reliability [38]. According to Bakeman et al. [39] for time-event data, the global kappa index based on time units ranged from 0.90 to 0.97 for all criteria, and the global kappa index based on events ranged from 0.72 to 0.87 for all criteria.

MMH Knowledge: MMH knowledge was measured with a instrument developed and based on the MMH-SsObserWork instrument [38]. This instrument consists of five criteria related to the main parts of the body involved in the MMH task (feet, knee joint, back, elbow joint), and the load position. Each criterion has its categories that describe the different positions that can be adopted. There are 16 categories in total. Categories are represented with pictures and a brief description. Workers had to indicate, for each criterion, which position was the most recommended to adopt during the MMH task by choosing a category. They had to indicate the most recommended for each MMH phase (lifting, carrying, and lowering). Hence, there were three identifications for each participant.

### 2.5. Data Management

To analyse MMH knowledge, we calculated the number of recommended positions identified for each criterion, and then the score change was computed in each criterion comparing the different moments (pre and post the first session; pre-first session and post-second session). In this sense, it was implied that a positive value meant an increase in the frequency of recommended positions identified.

To analyse MMH behaviour, the Lince software [40] was used to generate the observational record for each worker and session. The observational record provided information of time duration (frames and milliseconds) of each category for each criterion. This observational record was exported to MS-Excel. We did a recording of categories for each criterion, determining if they were placed in the recommended or non-recommended position, taking into account the literature review of MMH technique. As has been justified in previous works [38], the recommended positions established were: feet placed asymmetrically, one beside the load and the other behind it; knees in a semi-squat position (moderate flexion); a neutral back position at any front inclination of back; load placed closed to the body; arms extended or slightly flexed, and do not start or finish carrying phase while lifting and lowering phases are being carried out. The relative duration in which each criterion was found in the recommended position was calculated (time unit of recommended category as regards total duration).

The change scores for the different dependent variables (MMH knowledge and behaviour) were computed in order to control the hypothetical baseline effect.

### 2.6. Statistical Analysis

A basic statistical description had been made for each dependent variable, by group and time moment. The aim was to characterize the empirical distribution obtained. We assessed the assumption of normal distribution of variables using the Shapiro-Wilk test.

During the three-week follow-up, only 25 out of 31 participants from the SsObserWork group that initiated the follow-up answered the self-report questionnaire. Thirteen participants answered it between 11–15 days, 7 participants answered it between 6–10 days, and 5 participants answered it between 1–5 days. Due to the fact that there was irregular participation in answering the self-report questionnaire, we tested whether there were statistical differences in the dependent variables among those participants from the SsObserWork group who answered the self-report depending the number of days. For non-normal distributed variables, the differences were assessed using the Kruskal-Wallis test. For normally distributed variables, the differences were assessed using one-way analysis of variance.

The components effect on outcomes (MMH knowledge and behaviour) was assessed using independent samples *t*-test or Mann-Whitney U test on change scores, depending on the assumption of normality. The Mann-Whitney U test has been used when normality cannot be assumed. We specify the statistical test applied (*t*-test or Mann-Whitney) in Tables 2 and 3. Regarding the change score, a positive value indicates a change in the direction defined by the hypothesis.

Cliff’s δ statistics [41] were applied to assess component effect size for data that did not pass the normality test. The amount of effect sizes was interpreted as trivial (<0.147), small (between 0.147 and 0.33), medium (between 0.33 and 0.474), or strong (>0.474) [42]. For data that passed the normality test, effect sizes were examined using Cohen’s *d* statistics. Effect sizes were assumed as trivial (<0.20), small (between 0.20 and 0.49), medium (between 0.50 and 0.79), or large (>0.80) [43]. Analyses were performed using SPSS version 23 (SPSS Institute, Cary, NC, USA). However, R programme version 3.3.3 (R Core Team, R Foundation for Statistical Computing, Vienna, Austria) was used to analyse effect sizes. Statistical significance was set at *p* ≤ 0.05.

We conducted a supplementary analysis focused on delving into the temporal structure of employee behaviour, specifically with participants of the SsObserWork group who experienced an improvement in back position. The type of observational instrument and observational design that we used and adopted allowed to conduct a T-pattern analysis. This type of analysis allows to detect hidden or non-obvious temporal patterns in behaviour that are not always visible. Our aim was to detect T-patterns that involved a recommended back position, and we compared them between sessions of the SsObserWork group. From the observational record of each SsObserWork participant and session, we could detect the criteria co-occurrences during the MMH period (we called it a forward event). To detect temporal regularities in the order of event occurrences, we used the detection algorithm developed by Magnusson [44,45], and implemented in THEME^TM^ software (Patternvision Ltd., Reykjavík, Iceland). This detection algorithm first identifies significant (non-random) recurrences of any two events within a similar temporal configuration (critical interval) in real-time behavioural data and then proceeds to identify a hierarchical relationship with any other antecedent or subsequent events. This statistical method of detecting temporal patterns (T-patterns) of related behavioural events provides behavioural structures that may not be identifiable by traditional sequential methods [46,47]. Data was analysed using Theme 6.0. Default temporal patterns search parameters were used, an acceptable level of significance was set at 0.005, minimum occurrences at 3, and minimum percentage of samples (workers on this data) in which a pattern must occur, was set at 51%. The results have been validated by simulation, through randomization of data on five occasions, with acceptance only of patterns for which the probability of the randomized data coinciding with the real data is zero. The T-pattern differences between sessions were assessed using Pearson’s chi-squared test.

## 3. Results

### 3.1. Baseline Characteristics of Participants

Table 1 shows characteristics of the participants as regards demographic variables, presence of musculoskeletal disorders in the last 12 months, and the stage-of-change in physical exercise. In both groups, half of participants indicated that they had suffered lower back pain or discomfort in some time in the last 12 months. Additionally, Table 1 shows the mean and standard deviation of the self-perceived health status of participants and their work engagement. The two groups were very similar and with no statistically significant differences between them, suggesting that the randomization was successful.

### 3.2. MMH Knowledge

Table 2 presents the change scores (mean and standard deviation) of the number of recommended positions identified for each criterion that was carried out before and after the self-observation (in the SsObserWork group) and standard training (in the control group) of the first session. There were no significant differences between the groups in knowledge at baseline (first identification before starting the training). Significant differences between groups were found for the identification of recommended back position (U = −2.113, *p* = 0.035) before and after the first session, but no differences were found for the rest of criteria. The SsObserWork group reported better knowledge of back position that should be adopted in MMH compared to the control group (δ = 0.24) at the end of the first session. Moreover, Table 2 shows the scores changes (mean and standard deviation) of the number of recommended positions identified for each criterion that was carried out at the beginning of the first session and at the beginning of the second session. Significant differences between groups were also found for the identification of recommended back position (U = −2.032, *p* = 0.042), but there were no differences for the rest of criteria. The SsObserWork group still reported better knowledge of back position compared to the control group (δ = 0.29) after the follow-up period. As a result of irregular participation answering the self-report questionnaire, we also assessed the effect of answering it depending on the number of days on MMH knowledge. There were no statistical differences among participants of the SsObserWork group (see results in Appendix B, Table A2).

### 3.3. MMH Behaviour

Table 3 shows the score changes (mean and standard deviation) of the relative duration in which each criterion was placed in the recommended position during the MMH task that was performed in both sessions. There were no significant differences between groups in the relative duration in each criterion at baseline (before starting the training) except in the criterion elbow joints in which the control group showed higher relative duration in a recommended position rather than the SsObserWork group (U = −2.073; *p* = 0.038). We found statistical differences between the groups in the score changes of the back (*p* = 0.009), knee joints (*p* = 0.049), elbow joints (*p* = 0.021), and interaction criterion (*p* = 0.018), indicating that the SsObserWork group improved the MMH performance in these criteria. Focusing on the back position, after the first session and the follow-up period, the SsObserWork group increased the relative duration in which a neutral back position was adopted, compared to the control group who decreased the relative duration in the recommended position (δ = 0.42). As regards the knee joints, the SsObserWork group improved their overall position (moderate flexion at lifting and lowering phases, and extended position at the end of the lifting and at the beginning of the lowering phase). We also found that the SsObserWork group increased the relative duration of elbow joints in the extended and light flexed position. Finally, the SsObserWork group improved the interaction criterion, indicating that they avoided moving around with a back tilt position while they were carrying the load (interaction between lifting and lowering phases with the carrying phase). As a result of irregular participation in answering the self-report questionnaire, we also assessed its effect on the number of days on MMH behaviour. Non-statistical differences were found among participants of the SsObserWork group (see results in Appendix B, Table A3).

### 3.4. Supplementary Analysis

**Figure 3:**
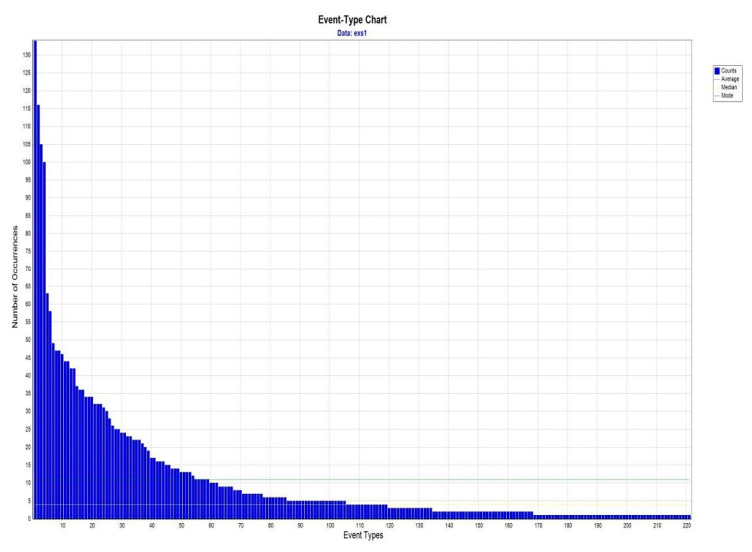
Distribution of event type during the first session.

**Figure 4:**
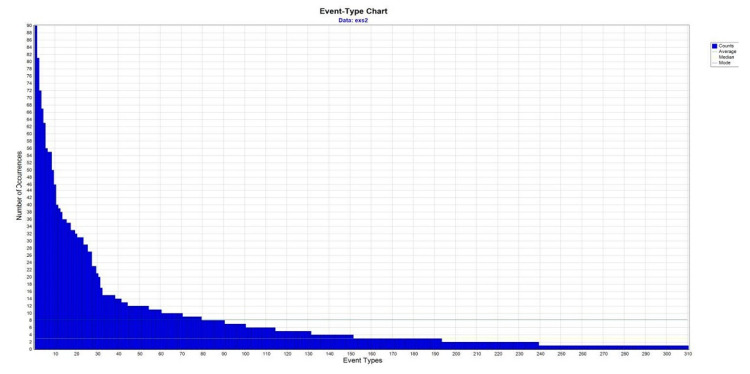
Distribution of event type during the second session.

As Table 3 shows, the SsObserWork group experienced an improvement in back position during the MMH task. We wanted to delve into the temporal structure of their behaviour, by detecting T-patterns that involved a recommended back position, and comparing it between sessions. Initially, we identified 221 types of events in the first session, and 310 in the second session. While the number of event types increased between sessions, their frequency of occurrence was found to be reduced (Figure 3 and Figure 4). Table 4 shows the percentage of event types that included the recommended back position. There was a significant increase of 7.5% in the types of events that included a neutral back position between the first and second session (χ2 = 37.769; gl = 1; *p* < 0.005). The types of events detected were submitted to the T-pattern analysis. Table 5 shows the number of T-patterns detected and the number of different T-patterns identified for each session. In the first session, a total of 3414 T-patterns were detected and there were 90 different T-patterns. In the second session, there was a reduction in significant T-patterns detected and a decrease in their diversification (446 T-patterns detected and 9 different T-patterns). On examining the parameters length, level, and occurrence of the T-patterns, it was observed that the reduction in the temporal structure of the behaviour in the second session occurs without reducing the variability in terms of the proportion of different patterns. Also noteworthy is the slight change in two aspects: (1) reduction in the complexity of the T-patterns after the intervention, and (2) an increase in their average occurrences. From the T-patterns detected in the first session, we established three indicators to focus on: T-patterns that included a recommended back position in one of their events, T-patterns that included a recommended back position in all of their events, and T-patterns that included a recommended position of back, elbow joints, and load (criteria related to the upper limb). Table 4 presents the percentage of T-patterns according to each indicator and the differences between sessions. The percentage of T-patterns in all three indicators were significantly higher in the second session compared to the first session (*p* < 0.0005).

## 4. Discussion

We hypothesised that the implementation of SSO, HFF, and intrinsic feedback would lead to improving employee knowledge and behaviour of the MMH technique. This hypothesis was confirmed. The SsObserWork group improved their knowledge and behaviour compared to the control group that received standard training.

As regards employee knowledge of the MMH technique, there was an increase in the recommended positions identified in both groups and comparing between times (before and after the first session, and comparing identifications at the beginning of the first session with ones conducted in the second session). The results showed that both interventions (SsObserWork and standard training) had a positive effect on employee knowledge, but knowledge of the recommended back position was significantly higher in the SsObserWork group, and this improvement was maintained between sessions. The SSO with the HFF allowed workers to focus their attention on detecting and discerning recommended and non-recommended back positions, compared to a general explication of how to perform an MMH task. The fact that workers could be observers of their own behaviour was more relevant because it had an emotional impact and gave them the opportunity to focus on qualitative aspects of their behaviour, identifying recommended and non-recommended positions.

As regards the control group, the results showed a decrease in the number of recommended identifications of back position between the identifications made at the beginning of the first session and with those made in the second session. In this case, the technician explained the instrument again to the control group in the second session, as was done with the SsObserWork group. This is why it is difficult to find an explanation for this result. However, we hypothesise that the standard training gave them general information on back position that could be retained for a short period (in the same session), but this information could be confused after a longer period. In fact, few studies have evaluated the intervention effect on knowledge of the MMH technique that could help reach a clearer conclusion. Additionally, some of these studies did not describe the questionnaire and some others used general questions about MMH [7].

Some studies observed that their intervention had an effect on the knowledge of MMH technique, but it did not have an effect on their behaviour [7]. The present study has not only observed effects on knowledge, but also on behaviour. The results showed that the SsObserWork group significantly increased the relative duration in which the back, knee joints, elbow joints and interaction between back tilt and move around were in recommended positions during the MMH task between the first and second session and comparing with the control group. On looking deeper into the results of the back position of the SsObserWork group, the T-pattern analysis showed a statistical increase in the events type that included a recommended back position, and this analysis allowed studying the change in the temporal structure of these events. After the intervention, it was observed that the temporal structure of behaviour was reduced, and there was a statistical increase in the percentage of patterns with recommended positions. These results suggest that the intervention not only had an effect on increasing recommended positions, but on also modifying the movement pattern in the MMH performance. Hence, the SSO, HFF, and intrinsic feedback contributed to adopting a neutral back, as well as recommended positions of knee joints and elbow joints during MMH tasks. These improvements are relevant because MMH training should not only focus on back but also on other body parts that are involved actively, and could effect the forces and posture of the back [48].

The SSO allowed workers to realize the need for a change in their MMH performance by active error detection, which was augmented with the HFF that directed their attention, something that is necessary because of so much information offered in the video. Watching oneself has an emotional impact that helps to attract the attention of the workers, which does not happen when theoretical information is given without any connection to them. In fact, when workers were observing themselves, they were astonished because they figured that they adopted recommended positions. It does not mean that they reacted negatively when they watched themselves, something that happened in the study of Linnerooth et al. [49], who associated the negative reactions of workers with non-self-observation effects. This is an issue that has to be considered because not everyone likes watching their own behaviour. Other studies have highlighted the positive effects of self-observation in other fields, such as improving teaching [16] and sports skill acquisition [13]. In the occupational health field, Taieb-Maimon et al. [17] observed that combining ergonomic training with self-modelling had more positive effects on workers’ postures than traditional ergonomic training, and the improvement lasted over time. Furthermore, Backåberg, Rask et al. [18], observed that self-observation promoted body self-awareness. In their qualitative study, they identified that participants pointed out the positive effects of describing verbally the body positions adopted at the beginning of the session [19]. As regards the results of Backåberg et al. [18,19], the present study complemented the SSO and HFF with promoting the intrinsic feedback. The aim of promoting the intrinsic feedback was to raise body self-awareness in order to make the employees capable of paying attention to their movements and self-correction, because after the training, they rely on their intrinsic feedback [26]. In this case, the HFF provided knowledge as regards ensuring that intrinsic feedback was being interpreted correctly. This suggests that technician feedback has to be provided after interpreting intrinsic feedback to require cognitive effort [20].

### Strengths and Limitations

The main strength of this study is the methodological complementarity by combining elements of the experimental design with those of observational methodology. This multi-method perspective allowed integrating data from an experimental and observational design, providing richer data to explain employee behaviour change after the intervention. The observational data obtained by using the MMH-SsObserWork instrument allowed characterizing the behaviour change from two perspectives: duration in recommended positions and T-patterns detection. As far as we know, this is the first study in occupational health field that assess behaviour change by implementing T-pattern analysis, which provides relevant information of how the training effects the pattern of the workers’ behaviour. Additionally, to our knowledge, this is the first study that assessed the effect of SSO, HFF, and intrinsic feedback as components of MMH training in the industrial sector, implemented during working hours.

As regards limitations, few participants of the SsObserWork group answered the self-report questionnaire during the follow-up period. Thus, this component was not implemented as expected due to the lack of compliance by the workers, even though we sent them reminders by text messages every day. Probably, the reason for the lack of follow-up of the self-report questionnaire was the effort required by the designed reporting system. The aim of the self-report questionnaire was to promote reactivity and collect data to identify whether the SsObserWork training was transferable. However, we did not identify significant differences between workers who completed the self-report questionnaire most days with those who did not complete it. The self-report questionnaire could have helped obtain information related to training transferability into their daily working life. This is essential to know if there is to be optimal feasibility and acceptability of the intervention and to identify barriers and facilitators to transfer it [4]. Therefore, we have to work on finding a better system to ensure worker compliance.

The next step is to assess transferability in their working and daily life, and to observe if the improvement in knowledge and behaviour will last over time. We are aware of the complexity and industriousness of the intervention’s approach, which is why our future aim is to tailor the training by focusing on key workers, such as older workers or supervisors. The objective will be focused on training them to be trainers in their professional and personal environments. Efficacy will be under well-controlled circumstances and will need to improve effectiveness in a real working-life situation. However, it is methodologically complex to implement and assess this type of training in a company if the requirement to provide behavioural evidence is maintained, as has been done in the present study.

## 5. Conclusions

We conclude that SsObserWork intervention, based on SSO, HFF, and intrinsic feedback, had a positive effect on improving knowledge and behaviour of the MMH technique, specifically on back posture. The combined use of SsObserWork components opens new possibilities for these participatory approaches, identifying the SsObserWork approach as a piece to integrate in multicomponent occupational interventions.

## Figures and Tables

**Table 1:** Baseline characteristics of participants in both study groups.

	SsObserWork Group	Control Group
Sex: *N (%)*		
Men	12 (38.7)	14 (46.7)
Women	19 (61.3)	16 (53.3)
Age: *N (%)*		
18 to 28 years	-	2 (6.7)
29 to 39 years	12 (38.7)	10 (33.3)
40 to 50 years	11 (35.5)	7 (23.3)
+50 years	8 (25.8)	11 (36.7)
Years working in the company: *N (%)*		
Less than one year	1 (3.2)	2 (6.7)
1 to 5 years	7 (22.6)	7 (23.3)
5 to 10 years	7 (22.6)	7 (23.3)
More than 10 years	16 (51.6)	14 (46.7)
Musculoskeletal disorders in the last 12 months: *N (%)*		
Suffered pain or discomfort at any body part	30 (96.8)	21 (70)
Suffered lower back pain or discomfort ^a^	16 (53.3)	15 (71.4)
Stage of change in physical exercise: *N (%)*		
Precontemplation	3 (9.7)	3 (10)
Contemplation	2 (6.5)	6 (20)
Preparation	14 (45.2)	12 (40)
Action	-	-
Maintenance	12 (38.7)	9 (30)
Self-perceived health status: *M (SD)*		
Physical Component Summary	50.69 (7.01)	49.47 (7.78)
Mental Component Summary	50.18 (5.89)	50.20 (6.07)
Work engagement: *M (SD)*		
Vigor	4.51 (1.11)	4.69 (0.95)
Dedication	3.90 (1.67)	3.97 (1.49)
Absorption	4.09 (1.36)	4.17 (1.44)
Total score	4.19 (1.26)	4.29 (1.16)

Abbreviations: M—Mean; SD—Standard Deviation. ^a^ Percentages obtained with respect to the participants who indicated having suffered pain or discomfort at any body part.

**Table 2:** Mean and standard deviation of score change of the frequency of recommended positions for criteria identified by the SsObserWork and control group, and differences between them.

	Before and after the First Session ^a^							At the Beginning of the First and the Second Session ^b^						
Outcome (Criteria Position)	SsObserWork Group		Control Group		Differences between Groups		Effect Size	SsObserWork Group		Control Group		Differences between Groups		Effect Size
	Mean	SD	Mean	SD	U ^c^	*p*-Value	δ ^d^	Mean	SD	Mean	SD	U	*p*-Value	δ
Feet	1.33	1.06	1.53	0.78	−0.514	0.607	−0.06	0.68	1.31	0.44	1.31	−0.635	0.525	0.09
Knees joint	0.37	0.85	0.73	1.08	−1.547	0.122	−0.20	0.25	0.97	0.63	1.36	−1.406	0.160	−0.20
Back	0.63	1.03	0.10	0.61	−2.113	0.035 *	0.24	0.25	1.21	−0.48	1.40	−2.032	0.042 *	0.29
Elbows joint	1.47	1.31	1.03	1.22	−1.482	0.138	0.22	1.11	1.26	0.52	1.42	−1.579	0.114	0.24
Load position	0.47	0.97	0.80	1.19	−0.988	0.323	−0.12	0.36	1.03	0.30	0.91	−0.010	0.992	0.00

^a^ 30 participants from the SsObserWork group and 30 participants from the control group were included in the analysis. ^b^ 28 participants from the SsObserWork group and 27 participants from the control group were included in the analysis. ^c^ Mann-Whitney U test. ^d^ Cliff’s δ statistics. * *p* < 0.05.

**Table 3:** Mean and standard deviation of score change (pre-post) of the relative duration in which each criterion was in the recommended position by the SsObserWork and control group, and differences between them.

	SsObserWork Group ^a^			Control Group ^b^			Differences between Groups		Effect Size
	Mean	SD	Median	Mean	SD	Median	U ^c^/t ^d^	*P*-value	δ ^e^/*d* ^f^
Feet	15.34	33.89	0.00	6.22	27.41	0.00	−1.884 ^c^	0.060	0.27 ^e^
Knees joint	12.69	24.42	9.79	1.45	15.04	−2.49	2.025 ^d^	0.049 *	0.55 ^f^
Back	7.21	16.37	3.72	−5.89	16.01	−6.78	−2.615 ^c^	0.009 **	0.42 ^e^
Elbows joint	33.58	38.33	11.29	9.11	31.13	4.04	−2.313 ^c^	0.021 *	0.37 ^e^
Load position	11.86	20.23	9.55	4.29	21.66	−2.72	−1.619 ^c^	0.105	0.26 ^e^
Interaction between back tilt and move around	5.10	7.53	2.87	3.08	17.00	0.17	−2.366 ^c^	0.018*	0.38 ^e^

^a^ 27 participants included in the analysis. ^b^ 26 participants included in the analysis. ^c^ Mann-Whitney U test. ^d^ Independent samples *t*-test. ^e^ Cliff’s δ statistics. ^f^ Cohen’s *d* statistics. * *p* < 0.05, ** *p* < 0.01.

**Table 4:** T-patterns detected that included recommended positions and differences between sessions.

	First Session		Second Session		Differences between Sessions	
	N	%	N	%	χ^2^	*p*-Value
Event types	221		310			
Event types with RBP		20.9		28.4	37.769	<0.0005 **
T-pattern detected	3414		446			
T-patterns included RBP		84		100	83.080	<0.0005 **
T-patterns (all events included with RBP)		14		100	1602.296	<0.0005 **
T-patterns included RBP, RLP, REJP		7		50	713.756	<0.0005 **

** *p* < 0.001; χ^2^—Pearson’s chi-squared test. Abbreviations: RBP—recommended back position; RLP—recommended load position; REJP—recommended elbow joint position.

**Table 5:** Descriptive statistics of the T-patterns detected in each session.

	First Session					Second Session				
	N	Mean	SD	Min-Max	%	N	Mean	SD	Min-Max	%
T-pattern different	90	.	.	.	.	9	.	.	.	.
Length	.	2.848	0.733	2–4	.	.	2.333	0.5	2–3	.
Level	.	1.778	0.667	1–3	.	.	1.333	0.5	1–2	.
Occurrences	.	37.933	10.351	23–84	.	.	49.556	10.725	37–64	.

Abbreviations: M—Mean; SD—Standard Deviation; Min-Max—minimum and maximum.

## Appendix A

**Table A1:** Description of the components, formative activities, and didactic material that make up the SsObserWork intervention.

**Components**	
Systematic self-observation (SSO)	After the MMH performance and recording, the SSO was done. It consisted of observing their own MMH performance by using a self-observation instrument that guides the observation and direct worker’s attention towards error detection or emphasise positive behaviours.
Hetero-observational feedback and feedforward (HFF)	It was provided during the SSO by the technician. It contributed to the worker’s direct attention towards qualitative information, such as whether what they do is recommended or not, and what and how to change (feedforward).
Self-report questionnaire	Through an ad hoc on-line questionnaire, workers self-reported aspects related to MMH tasks and physical exercise that they did every day (workplace and daily life) during the three-week follow-up. The questions were focused on frequency of MMH, implementation of technique learned, and performing of the physical exercises taught. The questionnaire had to be completed once a day by using a mobile phone. The aim was to complement the self-observation implemented during face-to-face sessions, by doing it every day.
Intrinsic feedback	Immediately after the MMH performance, workers had to recall and indicate how each body part was generally positioned during the MMH (for each phase) by using the self-observation instrument. The aim was to make workers aware of the importance of paying attention to the proprioceptive information and correcting oneself. Intrinsic feedback is also promoted during MMH practice.
Physical exercise	Three exercises were taught during the first session. These three exercises were chosen because are highly associated with the MMH movement pattern. These exercises train to adopt a neutral back and help to fit the body. The exercises were abdominal hollowing, hip hinge, and half -squat (at least 10 repetitions of each exercise had to be performed per day).
Motivational text messages	Motivational text messages were sent every day to their mobile phones and were used to reinforce and remind workers to adopt and implement what they had learned. There were two types of text messages: (a) Those used to motivate to do the three physical exercises. The message content was adapted to each worker according to their stage of change in physical exercise. Every day, the content was different. (b) Those used to remind workers to complete the self-report questionnaire. The text messages were sent every day in a strategic time of the day (when the worker was out of work) and a questionnaire link was attached in the text message.
**Formative Activities**	
MMH practice	Activity done in the first session. It was carried out after the SSO. Workers could practice and experience the MMH technique taught. Workers had to move a box and other things (e.g., a 5-litre bottle of water). By practicing the MMH technique, intrinsic feedback was potentiated and workers could identify benefits and resolve the barriers that were identified. It contributed to raise self-efficacy perception.
Physical exercise practice	The technician showed and taught how to perform each physical exercise. While workers had to perform each exercise, the technician corrected all the mistakes in order to make sure that workers would perform each exercise at home properly.
**Didactic Material**	
Self-observation instrument	It guided the observation and directed the worker’s attention towards each body part involved in the MMH task.
Website and informative triptych	Both sources provided all the information to eliminate any type of barrier related to forgetting how to do all exercises and the MMH technique. The website provided audio-visual information (videos), and on the other hand, the triptych provided written information with pictures.

## Appendix B

**Table A2:** Differences in the score change of the frequency of recommended positions for criteria identified by the SsObserWork group according to the number of days the self-report questionnaire was completed.

		Before and after the First Session				At the Beginning of the First and the Second Session			
Outcome (Criteria Position)	Groups Depending on Number of Days			Differences between Groups				Differences between Groups	
		Mean	SD	χ^2^	*P*-value	Mean	SD	χ^2^	*p*-Value
Feet	1 to 5 days	1.75	0.5	0.757	0.685	1.00	1.15	0.86	0.649
	6 to 10 days	1.28	0.95			1.00	1.29		
	11 to 15 days	1.33	0.98			0.42	1.44		
Knees joint	1 to 5 days	−0.25	0.50	2.942	0.230	-	-	0.48	0.787
	6 to 10 days	0.42	0.79			0.14	1.07		
	11 to 15 days	0.58	1.08			0.33	1.15		
Back	1 to 5 days	0.50	0.58	0.297	0.862	−0.55	0.50	1.32	0.517
	6 to 10 days	1.14	1.46			1.00	1.29		
	11 to 15 days	0.58	0.99			0.25	1.21		
Elbows joint	1 to 5 days	2.25	0.96	0.709	0.701	0–75	1.71	0.886	0.642
	6 to 10 days	1.14	1.34			0.86	1.21		
	11 to 15 days	1.17	1.53			1.25	1.22		
Load position	1 to 5 days	0.25	0.50	2.267	0.322	0.25	0.50	2.064	0.356
	6 to 10 days	0.58	1.16			−0.14	0.38		
	11 to 15 days	-	-			0.50	1.24		

*p* < 0.05; Kruskal-Wallis Test, χ^2^—Pearson’s chi-squared test.

**Table A3:** Differences in the score change (pre-post) of the relative duration in which each criterion was in the recommended position by the SsObserWork group according to the number of days the self-report questionnaire was completed.

Outcome (Criteria Position)	Groups Depending on Number of Days			Differences between Groups			
		Mean	SD	χ^2^	*p*-Value	F	*p*-Value
Feet	0 days	37.8	26.2	4.40	0.221		
	1 to 5 days	19.5	22.9				
	6 to 10 days	5.6	53.2				
	11 to 15 days	11.6	27.8				
Knees joint	0 days	6.4	22.7			0.178	0.911
	1 to 5 days	16.8	33.6				
	6 to 10 days	16.8	20.8				
	11 to 15 days	11.4	25.9				
Back	0 days	11.7	19.6	1.23	0.745		
	1 to 5 days	4.4	15.8				
	6 to 10 days	2.7	15.9				
	11 to 15 days	8.8	17.2				
Elbows joint	0 days	17.7	34.7	1.71	0.635		
	1 to 5 days	52.6	34.7				
	6 to 10 days	32.4	49.9				
	11 to 15 days	33.1	36.6				
Load position	0 days	18.1	17.9	0.88	0.831		
	1 to 5 days	12.3	15.5				
	6 to 10 days	16.4	31.9				
	11 to 15 days	7.7	16.7				
Interaction between back tilt and move around	0 days	7.1	8.2	1.93	0.587		
	1 to 5 days	3.0	6.4				
	6 to 10 days	2.3	5.0				
	11 to 15 days	6.4	8.8				

*p* < 0.05; Kruskal-Wallis test, χ^2^—Pearson’s chi-squared test; ANOVA one way, F—Fisher statistic.
